# Allostatic Load Is Linked to Cortical Thickness Changes Depending on Body-Weight Status

**DOI:** 10.3389/fnhum.2017.00639

**Published:** 2017-12-22

**Authors:** Jonatan Ottino-González, María A. Jurado, Isabel García-García, Bàrbara Segura, Idoia Marqués-Iturria, María J. Sender-Palacios, Encarnació Tor, Xavier Prats-Soteras, Xavier Caldú, Carme Junqué, Maite Garolera

**Affiliations:** ^1^Departament de Psicologia Clínica i Psicobiologia, Universitat de Barcelona, Barcelona, Spain; ^2^Institut de Neurociències, Universitat de Barcelona, Barcelona, Spain; ^3^Institut de Recerca Pediàtrica Hospital Sant Joan de Déu, Barcelona, Spain; ^4^Montreal Neurological Institute, McGill University, Montreal, QC, Canada; ^5^Departament de Medicina, Universitat de Barcelona, Barcelona, Spain; ^6^Institut d'Investigacions Biomèdiques August Pi i Sunyer, Barcelona, Spain; ^7^CAP Terrassa Nord, Consorci Sanitari de Terrassa, Barcelona, Spain; ^8^Brain, Cognition and Behavior Clinical Research Group, Consorci Sanitari de Terrassa, Terrassa, Spain; ^9^Unitat de Neuropsicologia, Hospital de Terrassa, Consorci Sanitari de Terrassa, Barcelona, Spain

**Keywords:** overweight, obesity, allostatic load, chronic stress, inflammation, cortical thickness, magnetic resonance imaging

## Abstract

**Objective:** Overweight (body mass index or BMI ≥ 25 kg/m^2^) and stress interact with each other in complex ways. Overweight promotes chronic low-inflammation states, while stress is known to mediate caloric intake. Both conditions are linked to several avoidable health problems and to cognitive decline, brain atrophy, and dementia. Since it was proposed as a framework for the onset of mental illness, the allostatic load model has received increasing attention. Although changes in health and cognition related to overweight and stress are well-documented separately, the association between allostatic load and brain integrity has not been addressed in depth, especially among overweight subjects.

**Method:** Thirty-four healthy overweight-to-obese and 29 lean adults underwent blood testing, neuropsychological examination, and magnetic resonance imaging to assess the relationship between cortical thickness and allostatic load, represented as an index of 15 biomarkers (this is, systolic and diastolic arterial tension, glycated hemoglobin, glucose, creatinine, total cholesterol, HDL and LDL cholesterol, triglycerides, c-reactive protein, interleukin-6, insulin, cortisol, fibrinogen, and leptin).

**Results:** Allostatic load indexes showed widespread positive and negative significant correlations (*p* < 0.01) with cortical thickness values depending on body-weight status.

**Conclusion:** The increase of allostatic load is linked to changes in the gray matter composition of regions monitoring behavior, sensory-reward processing, and general cognitive function.

## Introduction

Overweight (BMI ≥ 25 kg/m^2^) has become a major health problem worldwide; it affects up to 50% of the adult population in Western societies and it is a preventable risk factor for several medical conditions (World Health Organization, 2016). Caloric intake is mainly regulated by the homeostatic system (that is, the hypothalamus and brainstem structures) which promotes hunger or satiety responses via gut hormones and peptides (Dagher, 2009). However, the rewarding properties of food such as its odor and palatability can trigger approaching behaviors mediated by the hedonic or reward system (i.e., midbrain areas, striatum, and orbitofrontal cortex) even in the absence of hunger. This situation can be especially worrisome if top-down regulating systems (that is, cognitive-control areas such as the dorsolateral prefrontal cortex) fail frequently in regulating these urgent drives toward high-caloric food ingestion. Alterations within reward-processing and cognitive-control regions have been proposed as one of the possible mechanisms underlying pathological eating, or eating beyond caloric needs (Leigh and Morris, 2016). In addition, excess of weight and high-fat diets can be important sources of physiological stress as they promote chronic low-inflammation states detrimental for both physical and mental health (Castanon et al., 2014; Nguyen et al., 2014; Guillemot-Legris and Muccioli, 2017). Metabolic by-products characteristic of obesity augment the permeability of the blood-brain barrier and induce a sustained liberation of pro-inflammatory cytokines (e.g., interleukin-6, or IL-6), which ultimately increase the activity of the hypothalamic-pituitary-adrenal (HPA-) axis (Foss and Dyrstad, 2011).

Stress is a complex process resulting from an imbalance between one's resources and the demands of a given situation. It requires psychological, behavioral and physiological adaptations in order to overcome a defiant or threatening situation. This physiological modification, a process known as allostasis, includes significant and necessary changes in numerous biological systems (e.g., cardiovascular, metabolic, endocrine, and immune) to guarantee the energy boost needed during challenging scenarios (Kemeny, 2003). According to the allostatic load (AL) model (McEwen et al., 2015), the constant pushing of biological systems beyond their maximum capacity have a harmful cumulative effect that can lead to illness, or the so-called tertiary outcomes (e.g., hypercortisolemia, type II diabetes, psychiatric illness, and so on). Additionally, a recent study suggested that higher levels of circulating cortisol are related to an enhanced motivation for highly palatable food, greater abdominal fat accumulation and to an altered insulin metabolism (Jackson et al., 2017). As fight-or-flight responses consume a lot of energy, such changes in eating behavior and metabolism occur as a compensatory mechanism to restore energy reserves (Kemeny, 2003). Consequently, both hypercaloric ingestion and fat mass increasing might trigger inflammatory responses as well (Jastreboff et al., 2013; Leigh and Morris, 2016).

Neuroimaging findings from structural studies have evidenced gray matter reductions (Bobb et al., 2014) and cortical thinning (Marqués-Iturria et al., 2013; Veit et al., 2014) in regions of the prefrontal cortex associated with reward-processing and self-regulation (among other functions) in overweight subjects. According to stress-related neuroanatomical studies the prefrontal cortex has numerous adrenergic receptors, which made this region a target for chronic stress (Arnsten, 2009; Kremen et al., 2010; Leritz et al., 2011; Taki et al., 2013; Savic, 2015). As occurs in obesity and pathological eating, the settlement of abnormal stress responses also depends on blunted top-down regulating cortical areas (e.g., dorsolateral prefrontal cortex) on overly reactive limbic and subcortical structures (Leigh and Morris, 2016). In addition, posterior brain areas in temporal, parietal, and occipital lobes also show morphological alterations related to both overweight (Bobb et al., 2014; Veit et al., 2014; Kharabian Masouleh et al., 2016) and stress (Leritz et al., 2011; Taki et al., 2013). Some of these regions are ascribed to networks such as the dorsal-ventral attention, default mode, or cognitive-control networks. Although alterations in general health status, brain integrity, and cognition linked to both stress and overweight are well-documented separately, the relationship between the concept of AL and brain integrity has not been addressed to date, especially among overweight subjects. In fact, only two studies have assessed the detrimental effects related to an increase of AL in the cerebral cortex: the first showed total brain volume reductions in relation to AL increase in later life (Booth et al., 2015), and the second showed cortical thinning in both patients with schizophrenia and healthy controls (Chiappelli et al., 2017).

Along with the growing concern about excess weight as a body stressor, scientific interest in the concept of AL has increased in recent years. Current literature revolves around the hypothetical role of chronic physiological stress and inflammation during the onset of mental disorders, cognitive decline and dementia (Juster et al., 2010; Beckie, 2012; McEwen et al., 2015). An overactive HPA-axis can induce long-term damaging consequences for the CNS through the incremented flow of excitatory aminoacids (e.g., excitotoxicity), glucocorticoids (e.g., decreased neurogenesis), and/or by triggering inflammatory processes (e.g., gliosis). Cell signaling proteins secreted by the adipose tissue (e.g., leptin) could also activate the HPA-axis (Foss and Dyrstad, 2011; Abella et al., 2017; Guillemot-Legris and Muccioli, 2017). According to the AL frame, overweight subjects could be at an advanced stage of such model and endure large amounts of physiological stress, even in the absence of other cardiometabolic comorbidities as occur within our overweight-to-obese sample. For this reason, disentangling the relationship between stress and brain structure in overweight individuals is of considerable clinical value. The aim of this study is, therefore, to explore if there is a relationship between the whole-brain cortical thickness and the AL in a group of healthy overweight and lean adults. We expect to find a pattern of cortical thinning related to an increase in AL in both groups, and especially a pattern of greater severity among the overweight group.

## Materials and methods

### Subjects

The original sample consisted of one hundred and twenty-two adults recruited from public primary care centers belonging to the *Consorci Sanitari de Terrassa*. Eighty-four of them (43 obese individuals and 41 healthy controls) were already included in previous works (Ariza et al., 2012; García-García et al., 2013a,b,c, 2015; Marqués-Iturria et al., 2013, 2014, 2015). Since then, 38 new participants (29 overweight-to-obese and 9 lean subjects) have been recruited following the same procedure. Inclusion criteria were (1) being older than 20 years to guarantee a fully developed central nervous system (CNS), and (2) had a BMI higher than 18.5 kg/m^2^, which is the limit for underweight. The overweight group (*N* = 72) was formed based on a BMI higher than 25 kg/m^2^, following the World Health Organization's classification (World Health Organization, 2016). From the one-hundred and twenty-two included subjects, 25 of them (18 overweight-to-obese and 7 lean participants) met some of the exclusion criteria described below: (1) presence of past or present psychiatric illness (including addictive and/or eating disorders) or (2) any developmental, (3) neurological, or (4) systemic disorder (e.g., hyper or hypothyroidism, diabetes, cardiovascular disease). Participants were also ruled out because of (5) possible acute infection (C-reactive protein levels higher than 10 mg/L) or (6) meeting metabolic syndrome (MS) criteria (Alberti et al., 2009) (MS criteria are fully described in Supplementary Material section, Appendix A.1). Moreover, participants (7) with an estimated IQ below 85 (i.e., a scalar score lower than 7 in the WAIS-III vocabulary subtest) (Wechsler, 1999) were also excluded. Finally, the *Bulimia Inventory Test of Edinburgh* (BITE) (Henderson and Freeman, 1987) and the *Hospital Anxiety and Depression Scale* (HADS) (Zigmond and Snaith, 1983; Herrero et al., 2003) were used as exclusion criteria because high scores in these tests could suggest the presence of either an eating disorder (i.e., BITE score higher than 20) or anxiety-depression symptoms (i.e., HADS score higher than 11). The flow of included and excluded participants is detailed in depth in the subsequent SM section (Appendix A.2). Briefly, from this potential sample of 97 subjects, 20 overweight (of 54) and 14 lean subjects (of 43) declined to undergo a magnetic resonance imaging (MRI) acquisition of the head. Among the reasons for declining this phase of the study were the presence of claustrophobia, schedule incompatibilities, and/or being bearer of non-removable metallic objects (e.g., orthodontics). The final sample was composed by 34 overweight-to-obese subjects and 29 lean participants.

This study has been approved by the University of Barcelona's (CBUB) Institutional Ethics Committee, Institutional Review Board (IRB 00003099, assurance number: FWA00004225; http://www.ub.edu/recerca/comissiobioetica.htm). The research has been conducted in accordance with the Helsinki Declaration. Written informed consent was obtained from each participant prior to entry in the study.

### Allostatic load index

We calculated the AL index by selecting 15 stress biomarkers using the high-risk percentile procedure described in previous reviews (Juster et al., 2010; Beckie, 2012) and original articles (Chiappelli et al., 2017; Savransky et al., 2017) as a well-established way of measuring the concept. Additionally, leptin has been selected as a metabolic biomarker because its presence is directly proportional to the amount of adipose tissue, has modulating effects over blood pressure (Simonds et al., 2014) and induces the production of pro-inflammatory cytokines (e.g., IL-6) (Abella et al., 2017). Moreover, C-reactive protein (CRP), which is an unspecific indicator of an ongoing inflammatory process, served both as a biomarker and as exclusion criteria. High levels of CRP (>10 mg/L) may suggest acute infection, which may alter the values of other immunologic biomarkers (e.g., IL-6 or fibrinogen). Despite overweight subjects did not meet diagnostic criteria for cardiometabolic diseases by the time they entered the study, they were more likely to score higher in almost all biomarkers when compared to controls. Similarly to current studies in AL (Chiappelli et al., 2017; Savransky et al., 2017), the high-risk percentiles were based on the control group (*N* = 43) to avoid an overweight-driven floor effect. This sample presented similar characteristics to the overweight group (see Appendix B.1 in the SM section). Additionally, different cut-off points were set for each gender in those biomarkers (e.g., systolic arterial tension) that presented statistical differences (*p* < 0.05). Subjects in the high-risk percentile (i.e., greater than 75th, or below 25th in the case of HDL-cholesterol) obtained a binary score of “1” in that variable (or “0” if they did not exceed this cut-off point). The list of biomarkers and their cut-off points are shown below in Table 1. The AL index was the sum of all 15 biomarker dichotomous scores, with higher scores meaning higher allostatic overload (range 0–15). A prorated AL index has been conducted in participants with missing values. Additionally, we calculated an alternative index because reducting a variable to a bi-dimentional trait (i.e., 1 or 0) could result in a lose of information (see Appendix B.2 in the SM for more details).

**Table 1:** Allostatic load cut-off scores for individual biomarkers.

**Biomarker**	**Male**	**Female**	**Both**
Systolic arterial tension (mm Hg)	124.50	116.50	–
Diastolic arterial tension (mm Hg)	–	–	73
Glycated hemoglobin (%)	–	–	5.40
Glucose (mmol/L)	5.06	4.69	–
Creatinine (μmol/L)	90	70.25	–
Cholesterol (mmol/L)	4.21	4.88	–
HDL (mmol/L)	–	–	1.34
LDL (mmol/L)	–	–	3
Triglycerides (mmol/L)	–	–	0.86
C-reactive protein (mg/L)	–	–	0.93
Interleukin-6 (pg/mL)	–	–	1.65
Insulin (pmol/L)	–	–	52.02
Cortisol (nmol/L)	–	–	659.90
Fibrinogen (g/L)	3.07	3.32	–
Leptin (ng/mL)	4.80	20.30	–

*mm Hg, millimeters of mercury; cm, centimeters; mmol/L, millimoles per liter; μmol/L, micromoles per liter; mg/L, milligrams per liter; pg/mL, picograms per milliliter; nmol/l, nanomoles per liter; g/L, grams per liter; ng/mL, nanograms per milliliter*.

### Image acquisition

Overweight (*N* = 34) and lean (*N* = 29) participants underwent MRI on a 3T MAGNETOM Trio (Siemens, Germany), performed at the *Institut d'Investigacions Biomèdiques August Pi I Sunyer* (IDIBAPS) from the *Hospital Cl*í*nic* de Barcelona with the following parameters: repetition time 2,300 ms; echo time 2.98 ms; inversion time 900 ms; 240 slices, field of view 256 × 256 mm, 1 mm isotropic voxel in order to obtain high resolution T1-weighted MPRAGE 3D scans.

### Data processing

T1-weighted images has been processed using FreeSurfer (v.5.3) pipeline by default (i.e., recon-all). Briefly this step includes skull stripping (Ségonne et al., 2004), motion correction and T1 averaging (Reuter et al., 2010), bias-field correction (Sled et al., 1998), and tessellation of gray/white matter tissue. Representations of cortical thickness has been calculated as the closest distance from the gray/white boundary to the gray/CSF boundary at each vertex on the surface (Fischl and Dale, 2000). The surface was smoothed using a circularly symmetric Gaussian kernel with a full-width at half maximum of 15 mm. The post-processing outputs for each subject were examined visually to ensure processing accuracy. Manual editing was performed when required to improve pial surface reconstruction.

### Statistical analysis

Age, gender, IQ estimation (i.e., WAIS-III), and other sociodemographic (i.e., years of education, family income, professional level), psychological (i.e., HADS) and behavioral variables (i.e., smoking, drinking) were analyzed using Student's *t*-test and chi-square test (bootstrap 1,000 iterations) with IBM SPSS Statistics (v.23) to ensure that there were no significant differences (*p* ≥ 0.05) between groups. Regarding the imaging analyses, global thickness (in mm) brain measures (i.e., left and right hemisphere mean cortical thickness) were extracted to conduct group comparisons (i.e., ANOVA, *t*-test) in SPSS. Whole-brain analyses has been performed using the general linear modeling (GLM) implemented in the FreeSurfer's Qdec tool. The age, years of education, and gender were set by default as nuisance factors to avoid their potential biasing influence over brain morphology. We tested for group differences at the vertex-wise level in whole-brain cortical thickness in each hemisphere. The relationship between AL and cortical thickness was explored with linear regression analysis. This model included cortical thickness as the dependent factor and the AL index as the independent factor. The waist circumference (WC) was also included as nuisance factor in this model to assess this association independently of the effects of body weight by itself (Sahakyan et al., 2015). We first explored for associations in the entire sample (*N* = 63), and then we addressed the possibility of a group effect over this relationship (i.e., comparing the correlations coefficients of overweight and leans). All results were corrected for multiple comparisons using a precached Monte-Carlo null-Z Simulation (10,000 repetitions) with a cluster-wise corrected *p*-value (CWP) of <0.01 for statistical significance. The clusters that remained significant were reported according to the Desikan's atlas (Desikan et al., 2006) in MNI305 coordinates. Mean thickness (mm) of significant clusters were extracted for plotting results.

## Results

We found no significant differences between groups in sociodemographic, cognitive (i.e., IQ estimation), psychological (i.e., HADS), or behavioral data (i.e., smoking, drinking). As expected, groups statistically differed in BMI [*t*_(61)_ = −9.774, *p* < 0.001, BCa 95% −11.09~−7.30], WC [*t*_(61)_ = −8.212, *p* < 0.001, BCa 95% −26.18~−16.64], and AL index [*t*_(61)_ = −5.117, *p* < 0.001]. The results of this variables are available below in Table 2.

**Table 2:** Variables of interest of overweight and lean participants.

	**Overweight (*****N*** = **34)**		**Lean (*****N*** = **29)**	
	**Mean (SD)**	**Range**	**Mean (SD)**	**Range**
Age	31.79 (6.10)	21–40	30.07 (6.21)	21–40
Years education	13.76 (2.69)	10–20	14.62 (2.21)	10–18
IQ estimation	12.00 (2.16)	8–17	11.93 (1.83)	7–15
Gender (F/M)	21/13		15/14	
Smoker (yes/no)	10/24		6/23	
Drinker (yes/no)	17/17		18/11	
HADS anxiety	4.12 (2.41)	0–9	4.55 (2.97)	0–10
HADS depression	1.97 (2.17)	0–7	1.41 (1.57)	0–5
BMI (kg/m^2^)^*^	31.39 (5.02)	25.20–49.69	22.35 (1.82)	19.00–24.99
WC (cm)^*^	99.52 (13.26)	82–137	77.96 (6.70)	67–92
AL Index^*^	6.78 (2.64)	2–12	3.56 (2.29)	0–9
**FAMILY INCOME IN EUROS PER MONTH (FREQUENCY)**				
300–899	0		0	
900–1,499	4		5	
1,500–2,099	15		9	
2,100–2,699	8		4	
>2,700	6		10	
Don't know / Don't answer	1		1	
**PROFESSIONAL LEVEL (FREQUENCY)**				
Non-skilled	3		1	
Skilled manual	10		5	
Administrative	8		5	
Intermediate	5		6	
Professional	5		6	
Don't know / Don't answer	3		6	

* *p < 0.05, IQ estimation, Intelligence Quotient estimation (WAIS-III); F, female; M, male; BMI, body mass index (kg/m^2^); WC, waist circumference (cm); HADS, Hospital Anxiety and Depression Scale; SD, Standard deviation*.

**Figure 1:**
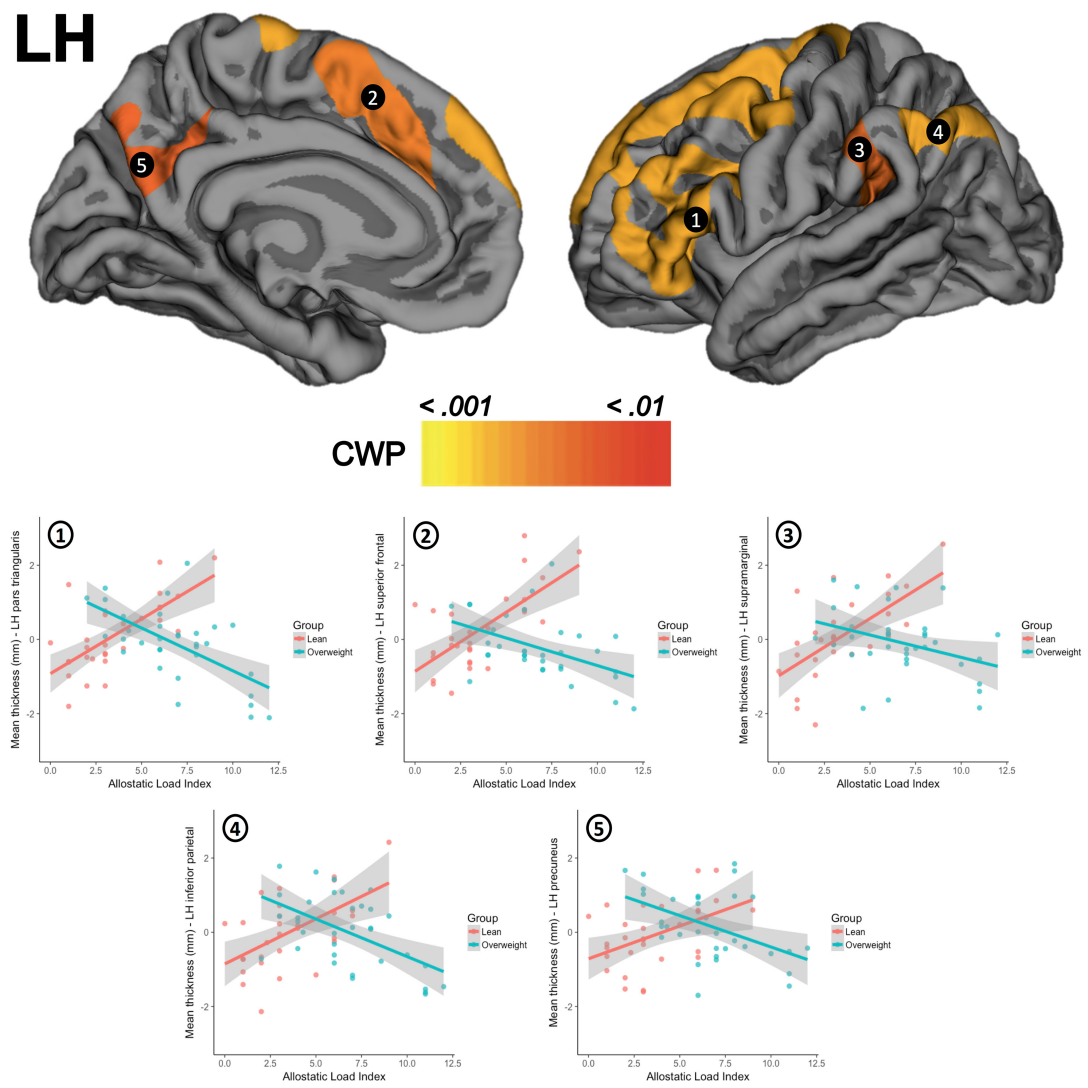
The upper row shows the interactions between groups for clusters in the left hemisphere (LH) in (1) pars triangularis, (2) superior frontal gyrus, (3) supramarginal gyrus, (4) inferior parietal cortex, and (5) precuneus. The second row presents scatterplots of the interaction between groups (overweight/blue, lean/red) for the standardized residuals of mean cortical thickness (Y-axis) and AL index (X-axis). CWP, cluster-wise corrected *p*-value.

**Figure 2:**
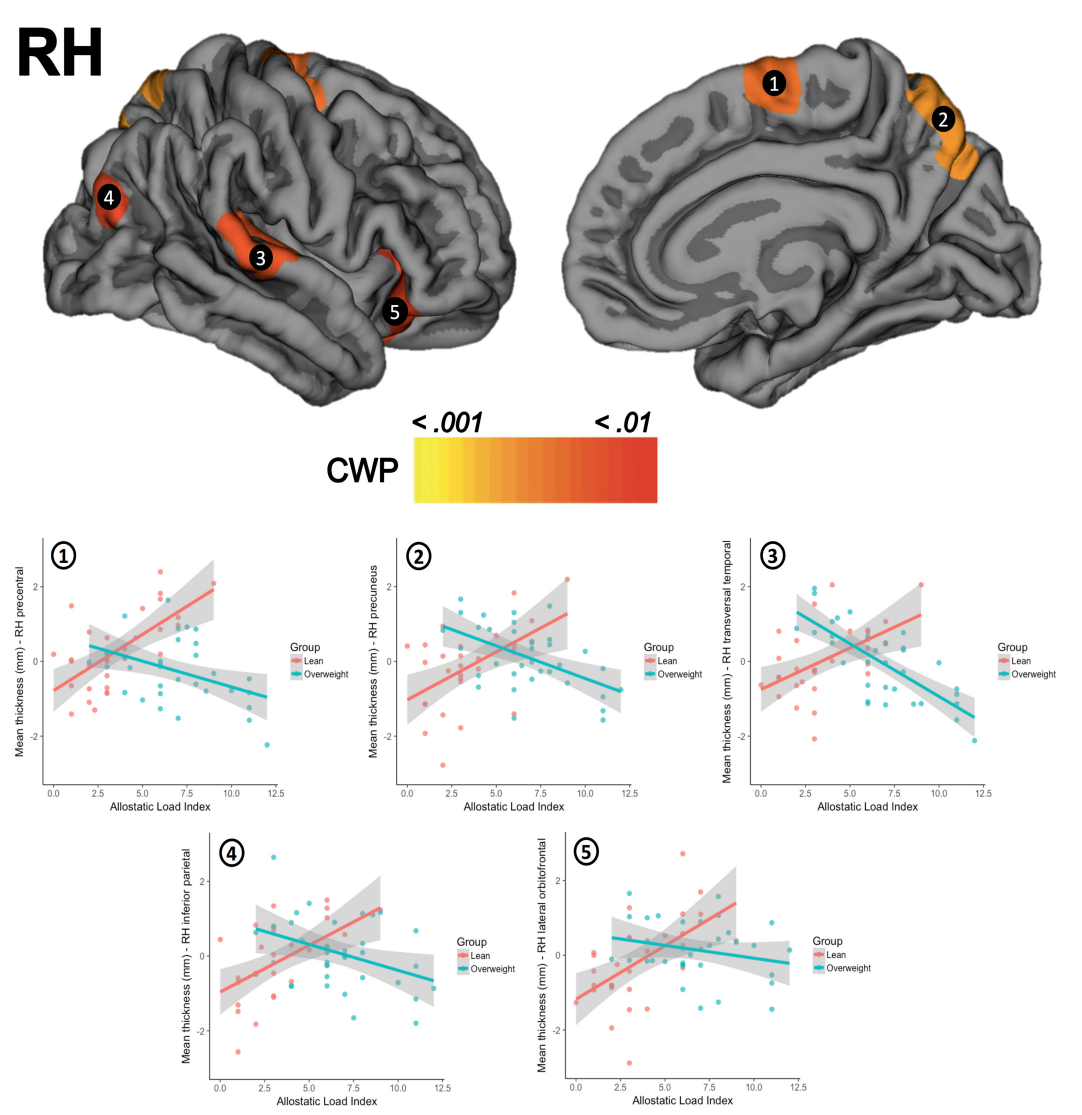
The upper row shows the visual interactions between groups for clusters in the right hemisphere (RH) in (1) precentral gyrus, (2) precuneus, (3) transversal temporal gyrus, (4) inferior parietal cortex, and (5) lateral orbitofrontal cortex. The second row presents scatterplots of the interaction between groups (overweight/blue, lean/red) for the standardized residuals mean cortical thickness (Y-axis) and AL index (X-axis). CWP, cluster-wise corrected *p*-value.

There were no significant differences for global thickness measures as shown in the SM section (Appendix C.1.). The whole-brain vertex-wise comparisons between groups revealed a significant difference in cortical thickness in the left superior frontal gyrus (X = −18.1, Y = 17.3, Z = 52.8; size = 1920.55 mm^2^, mean thickness in leans = 2.70 mm ± 0.13 and mean thickness in overweight = 2.53 mm ± 0.13, Z-value = 4.349, CWP < 0.001) and in the right superior frontal gyrus (X = 8.1, Y = 46.0, Z = 22.6, size = 2072.66 mm^2^, mean thickness in leans = 3.02 mm ± 0.14, mean thickness in overweight = 2.87 mm ± 0.16, Z-value = 4.132, CWP < 0.001). When tested for linear regressions in FreeSurfer's GLM within the whole sample (*N* = 63), there were no significant relationship between the AL index increase and the thickness of the cortical mantle. However, the results showed a significant interaction when this association was compared between groups. Overweight subjects showed lower cortical thickness as the AL index increase (i.e., thinning), while lean participants demonstrated greater thickness (i.e., thickening) as this AL score increased. This interaction was found in five clusters in the left hemisphere, with their maximum peak of intensity in the pars triangularis (CWP < 0.001), superior frontal gyrus (CWP < 0.001), supramarginal gyrus (CWP < 0.001), inferior parietal cortex (CWP < 0.001), and the precuneus (CWP = 0.001). An interaction was also significant on the right hemisphere and followed the same trend in clusters located in the precentral gyrus (CWP < 0.001), the precuneus (CWP < 0.001), the transversal temporal gyrus (CWP = 0.002), the inferior parietal cortex (CWP = 0.004), and the lateral orbitofrontal cortex (CWP = 0.005). Location, size, and coordinates of these interactions are shown in the SM section (Appendix C.2). Visual maps of cortical thickness patterns and the mean thickness standardized residuals (i.e., regressing out the effects of age, years of education) of each cluster are depicted in Figures 1, 2. Additionally, the results of the analysis conducted with the alternative AL index (i.e., factor reduction) and the visual representations of the overlapping results for both group comparisons and group interactions, are available in the SM section (Appendixes D.1, D.2, respectively).

## Discussion

We addressed the relationship between AL and cortical thickness in a sample of healthy overweight-to-obese adults and lean controls with similar characteristics. Few studies to date have focused on linking the AL and the brain integrity beyond psychiatric (Chiappelli et al., 2017; Savransky et al., 2017) or elderly samples (Booth et al., 2015). Given the association between overweight and physiological stress/inflammation (Foss and Dyrstad, 2011; Castanon et al., 2014; Nguyen et al., 2014; Guillemot-Legris and Muccioli, 2017; Jackson et al., 2017), assessing the relationship between the stress and brain integrity is relevant with a view on prevention and early medical interventions (e.g., mental health wellness, healthy aging, and successful weight loss). First, in the present study overweight participants showed lower cortical thickness compared to controls in two clusters located in left and right superior frontal gyrus, as previously described in Marqués-Iturria et al. (2013). Second, and regarding to the main objective of this study, overweight subjects proved have higher AL indexes than lean controls. The AL index increase did not show a relationship with the cortical thickness when included the entire group in the analyses. However, when we examined the presence of possible group interactions, we observed a pattern of cortical thinning in overweight and cortical thickening in leans in relation to AL increase. This interaction occurred in several bilateral anterior (frontal and prefrontal cortex) and posterior cortical regions (temporal and parietal cortex). Some of these areas are part of networks involved in monitoring behavior (including eating behavior), sensory-reward processing and support basic cognitive abilities (e.g., memory, attention, etc.).

The lack of significant results in the entire sample suggests in first place that the relationship between AL and cortical thickness may not follow a linear trend. Rather, our results suggest that this relationship may be modulated by body-weight status in a complex way. Moreover, although we expected that the AL increase would be linked to negative neurological outcomes (e.g., cortical thinning) independently of the group, the relationship between AL and cortical thickness did not behave as expected for lean subjects. A cortical thickening pattern has also been described in very early stages at the onset of major depressive disorder (Qiu et al., 2014) and schizophrenia (van Haren et al., 2011), during presumably an ongoing inflammatory process. According to Qiu et al. (2014), while cortical thinning may suggest dendritic retraction, cortical thickening may be a sign of glial activity promoted by pro-inflammatory cytokines in order to prevent neuronal degeneration by increasing neurotrophic factor release (e.g., brain-derived neurotrophic factor, or BDNF). This is, in lean subjects, an engaged HPA-axis and the liberation of pro-inflammatory cytokines induced by a challenging situation could be linked to cortical thickening (e.g., gliosis, neurotrophin signaling). Then, if this response does not revert and escalates (i.e., as it likely does in overweight subjects), it could be related to negative outcomes in the brain (e.g., dendritic retraction, neuronal degeneration, etc.). Our findings may represent the transition from allostasis (i.e., a natural response to a challenge) to allostatic load (i.e., a failed response to a repeated threat). Moreover, chronic-stress and/or adiposity (and its metabolic by-products, such as leptin, LDL cholesterol or triglycerides) could affect the functionality (e.g., hypertension) and structure of blood vessels (e.g., atherosclerosis). This, beyond being an important risk factor for cerebrovascular disease by itself, it can impair the glucose and oxygen transportation into the brain (Kemeny, 2003), which it might be as well another potential insult for neuronal integrity. The nature of the challenging situation (i.e., internal or external) and the causality between chronic-stress and overweight cannot be tackled with this design and statistical analysis. Nevertheless, our results showed that the increasing of AL index is linked to cortical changes whose pattern (i.e., thinning or thickening) depends on body-weight status.

We found a pattern of cortical thinning in overweight subjects and cortical thickening in controls related to an AL index increase in bilateral frontal and prefrontal cortex. Specifically, we observed changes in bilateral insula, precentral, lateral orbitofrontal and superior frontal gyrus, and in the left middle (rostral and caudal middle frontal) and inferior (pars orbitalis, pars opercularis, and pars triangularis) frontal gyrus. A similar pattern of results (especially with regards to middle and inferior frontal gyrus) have also been reported by Chiappelli et al. (2017) in their study of AL and schizophrenia. Moreover, volume reductions of these areas have been described in obese subjects when compared to leans, or when assessing the relationship between BMI and/or WC increase and gray matter integrity (Bobb et al., 2014; Kharabian Masouleh et al., 2016; Janssen et al., 2017) or cortical thickness (Marqués-Iturria et al., 2013). Some of these regions have also been reported to be affected during stress situations (Savic, 2015), or in relation to stress biomarkers (Kremen et al., 2010; Leritz et al., 2011; Zhang et al., 2016). The pars opercularis is a key region for the integration of sensory-hedonic properties of food (e.g., taste, texture, palatability) given its involvement along with the anterior portions of the insula in the primary gustatory cortex (Kumar et al., 2016). Orbitofrontal regions are known as a second gustatory cortex and for their role in reward-processing, a function affected in overweight (García-García et al., 2013a,c, 2014, 2015; Marqués-Iturria et al., 2015) and stress (Porcelli et al., 2012). Dorsolateral (i.e., middle and superior frontal gyrus) and ventrolateral portions (i.e., inferior frontal gyrus) of the prefrontal cortex are involved in regulating behavior through executive functions such as inhibitory control. The ability to suppress automatic responses and replace them with more appropriate ones seems to be altered in both overweight (Smith et al., 2011) and stress (Sandi, 2013). Finally, medial portions of the prefrontal cortex (i.e., medial superior frontal gyrus) are involved in decision-making and motivation, which also appear to be affected in overweight (Smith et al., 2011) and stress (Sandi, 2013). In coherence with Marqués-Iturria et al. (2013), dorsal and medial portions of both left and right superior frontal gyrus showed cortical thickness reductions in the overweight participants when compared to lean controls. Obesity status (overweight vs. lean) and AL seemed to share variance in the left superior frontal gyrus. However, this region remained significant after controlling for the effects of body weight in the linear regression analysis.

Our results showed dimorphic associations between cortical thickness and higher AL indexes in overweight and controls in bilateral superior temporal gyrus. Besides auditory and language processing, superior temporal gyri have also been associated along with the insula with satiation signal processing (Kroemer et al., 2013) and the ventral-attention network (Vossel et al., 2014). The temporal lobe also has been related to learning and memory processes. Alterations of memory performance are usually reported in both obesity (Higgs, 2015) and stress (Sandi, 2013). The vulnerability of this structure has been linked to adiposity (Veit et al., 2014; Kharabian Masouleh et al., 2016), stress exposure (Leritz et al., 2011; Taki et al., 2013), and AL increase (Chiappelli et al., 2017) as well.

We also observed a group-dependent pattern of cortical changes linked to higher levels of AL index in bilateral precuneus, inferior and superior parietal cortex, and paracentral and supramarginal gyrus. Some of these dorsal regions (i.e., supramarginal gyrus, inferior, and superior parietal cortex, etc.) exhibit a high degree of connectivity with dorsolateral prefrontal areas, together forming what is known as the cognitive-control or frontoparietal network. Along with inhibitory control, working memory is an executive function usually associated with this network (Darki and Klingberg, 2014), and it is involved in keeping information available when it is no longer perceptible. Working memory impairments have been linked to both stress (Sandi, 2013) and overweight (Higgs, 2015). It is believed that lack of access to our long-term goals (e.g., having an online record of caloric intake during the day, maintaining a daily healthy food consumption or not eating beyond caloric need) when required may contribute to bad dietary choices and overeating (Higgs, 2015). Other works have also associated gray matter reductions in dorsal parietal regions with adiposity (Bobb et al., 2014; Kharabian Masouleh et al., 2016) and stress (Leritz et al., 2011). Finally, a ventral region such as the precuneus is considered an important hub in the default mode network (DMN). The DMN (precuneus, posterior and anterior cingulate cortex, middle temporal gyri, and inferior parietal and medial prefrontal cortex) is related to self-referential information processing, and is engaged during “wakeful rest” when we think about others or ourselves. Structural modifications in this region have been consistently reported in several mental disorders (Anticevic et al., 2012) and stress (Leritz et al., 2011), but not in obesity (García-García et al., 2013b). However, the precuneus overlaps with reward-salience processing regions, a network which is affected in obesity (García-García et al., 2013a,b; Horstmann et al., 2015; Marqués-Iturria et al., 2015). In the study of Chiappelli et al. (2017), the increase of AL was related to decreases in the cortical thickness of parietal regions as the paracentral and postcentral gyrus, the inferior and superior parietal cortex, and the precuneus, similar to the results that we observed with overweight participants.

Some limitations emerge when it comes to the methodology under the AL measurement. Theorists have suggested that the AL model can provide an interesting and holistic perspective to explain the onset of several mental illnesses. In the present study, we assessed the AL following the most used method in the literature, which is based on high-risk percentiles and the sum of dichotomous scores. With this, we facilitate the replication of our results. However, it would help further research the fact of determining the normal (i.e., in absence of developed comorbidities) distribution of stress biomarkers based on large population studies accounting for age, gender, and socio-economic status. Since we excluded participants with medical pathologies, the use of clinical cut-off scores was not feasible. At least 9 out of 15 of the stress biomarkers used for this study referred to metabolic parameters. Because of this, it could be also reasonable to state that our results might be partially driven by the effect of metabolic variables. The AL concept may be confounded with MS, especially with the number of metabolic biomarkers we used in our work. However, the cortical thinning attributed to MS are mostly explained by the increase of the WC (Schwarz et al., 2017), which we controlled for its effects in all of our analyses. In this vein, and as the MS is usually referred to as a pre-stage for type II diabetes, the allostatic overload could be interpreted as a previous step for MS. Although more research is needed, the AL proved be useful for early detection of structural changes in young adults with a non-clinical status. As aforementioned, the exploratory nature, the cross-sectional design and the type of analysis performed in this study unfortunately do not allow concluding upon causality. Chronic stress may be a risk factor for weight gain; just as overweight may be an important source of chronic stress (Foss and Dyrstad, 2011). Chronic-stress increases the appetite for high-caloric food to restore energy reserves, yet modern stressors do not usually threat physical integrity nor require physical performances (i.e., fight-or-flight responses) as they once did. On the other hand, the fat tissue is capable of induce inflammation responses (in fact, early stress responses are originally intended to facilitate the vascularization of the increasing fat mass), which stresses the organism. Further works should focus on disentangling this issue, especially since not all chronically stressed people crave for highly palatable food and/or eat uncontrollably and gain weight. It is possible that when stressed, some people may involuntary increase their physical activity (e.g., augmented leg shaking and/or wandering around home/workplace), or change their habits in an overcompensating fashion (e.g., eating healthier, engaging in sport activities, etc.). Hence, psychological (e.g., personality traits, resilience, coping strategies), genetic (e.g., *5-HTT, BDNF, FTO*), behavioral (e.g., diet, smoking, exercise, sleep quality), socioeconomic and environmental (e.g., family income, education, neighborhood, exposure to contaminants) factors should be explored in order to identify phenotypes in which chronic stress predisposes to obesity development and vice versa. The sample size is another important limitation that makes difficult to generalize the results obtained. Our exclusion criteria are very strict in order to ensure that the observed results are not due to the presence of confounding variables (e.g., psychological distress, metabolic syndrome, or acute infection). On a similar note, the focus on overweight participants has a special clinical relevance, since this population does not necessarily show cardiometabolic comorbidities that are frequent in severe stages of obesity. This could facilitate the isolation of the effect of excess of weight. We encourage future works with bigger samples to replicate our findings. On the contrary, a strength that we would like to highlight is the use of samples with similar characteristics: the allostatic load cut-off points may vary according to gender, age and the socioeconomic and personal situation of each individual. Having matched groups ease the extrapolation of cut-off points for further investigations with similar groups. Additionally, and to the best of our knowledge, this is the first study approaching the AL concept in healthy overweight adults. Overweight is an increasingly prevalent condition that requires new therapeutic approaches to stop its progression and its associated complications.

In conclusion, our study suggests the existence of a dimorphic (i.e., thinning and thickenning) and detrimental relationship between AL increase and the morphology of brain structures that supervise behavior (e.g., inhibitory control, working memory), process the satiating signals and reward-hedonic properties of food (e.g., motivation/craving, satiety) and enable correct cognitive functioning (e.g., attention, memory). These brain regions are necessary to participate in healthy habits crucial for psychological and physical well-being. Early interventions in the general population may prevent the effects of chronic stress on the brain that can lead to serious potential comorbidities.

## Author contributions

JO-G, MJ, IG-G, BS, IM-I, CJ, and MG: all provided substantial contributions either to the conception and design of the study, analysis, and/or interpretation of the results; JO-G, IG-G, IM-I, XP-S, MS-P, and XC: also participated in data acquisition (subject recruitment, medical, and neuropsychological evaluation, MRI acquisition); Additionally, all authors critically revisited the work, approved its final version for publishing, and agreed to be accountable for all aspects of such work.

### Conflict of interest statement

The authors declare that the research was conducted in the absence of any commercial or financial relationships that could be construed as a potential conflict of interest.
