# Supplementary material for: Allostatic Load Is Linked to Cortical Thickness Changes Depending on Body-Weight Status
Source: Front Hum Neurosci. 2017 Dec 22;11:639. doi: 10.3389/fnhum.2017.00639 (PMC5770747; doi:10.3389/fnhum.2017.00639)
Supplement: Supplementary file 1 [file DataSheet1.docx]

1. **SUPPLEMENTARY MATERIAL**

| Appendix A.1 - Metabolic syndrome criteria.  At least three (3) of the presented above: | |
| --- | --- |
| **Measure** | **Cut-off point** |
| **Waist**  **circumference** | Male: 94 cm  Female: 80 cm |
| **Triglycerides** | 150 mg/dL (1.7 mmol/L) |
| **High density**  **lipoprotein**  **(HDL-C)** | Male: < 40 mg/dL (1.0 mmol/L)  Female: < 50 mg/dL (1.3 mmol/L) |
| **Arterial**  **pressure** | Systolic: 130 mm Hg  Diastolic: 85 mm Hg |
| **Fasting**  **glucose** | 100 mg/dL |
| cm = centimeters, mg/dL = milligrams per deciliter  mmol/L= micromoles per liter, mm Hg = millimeters of mercury | |


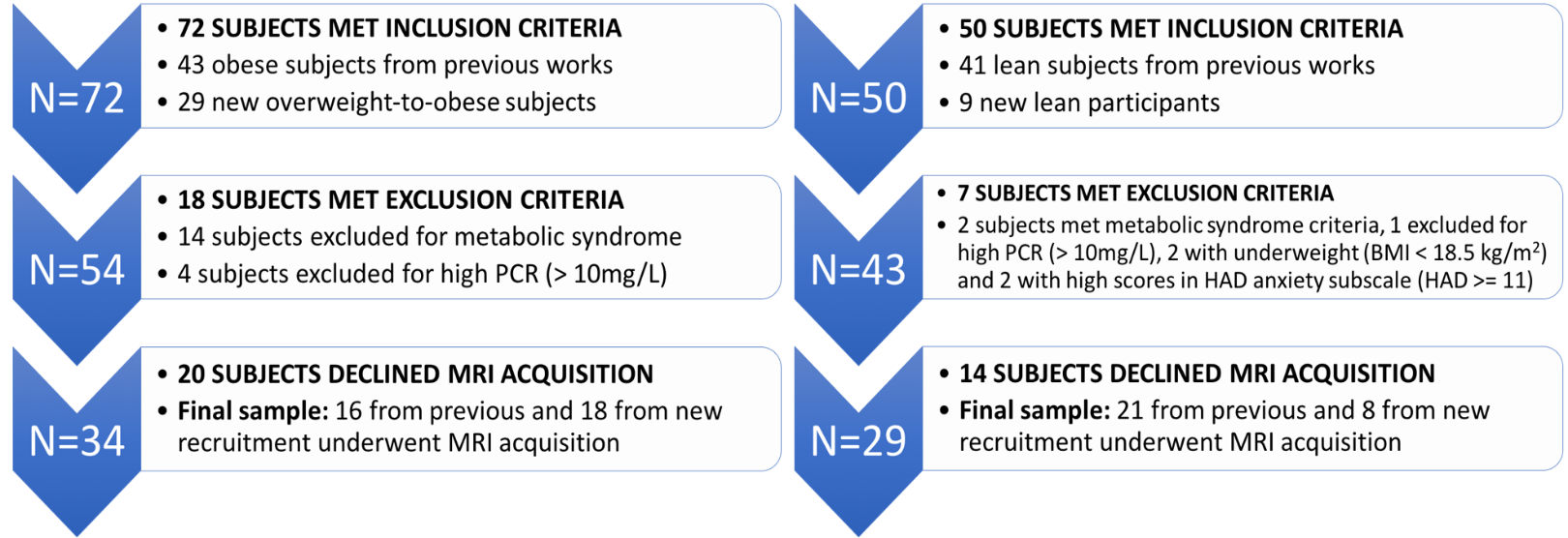


Appendix A.2. Flow of included and excluded participants

Appendix B.1. – Caractheristics of the normative group served for the cut-off calculation

|  | **Lean (N=43)** | |
| --- | --- | --- |
|  | **Mean (SD)** | **Range** |
| Age | 30.44 (6.03) | 21 – 40 |
| Years education | 14.12 (2.41) | 9 – 18 |
| IQ estimation | 11.50 (1.95) | 7 – 15 |
| Gender (F/M) | 26/17 | |
| Smoker (yes/no) | 9/34 | |
| Drinker (yes/no) | 24/19 | |
| HADS anxiety | 4.47 (2.80) | 0 – 10 |
| HADS depression | 1.26 (1.60) | 0 - 6 |
| BMI (kg/m^2^) | 21.99 (1.76) | 18.59 – 24.99 |
| WC (cm) | 76.05 (6.95) | 61 – 92 |
| **Family income in euros per month (frequency)** | | |
| 300-899 | 1 | |
| 900-1499 | 7 | |
| 1500-2099 | 13 | |
| 2100-2699 | 7 | |
| >2700 | 13 | |
| Do not know / do not answer | 2 | |
| Non-skilled | 4 | |
| Skilled manual | 5 | |
| Administrative | 8 | |
| Intermediate | 7 | |
| Professional | 9 | |
| Do not know / do not answer | 10 | |
| IQ estimation = Intelligence Quotient estimation, F = female, M = male, BMI = body mass index (kg/m^2^), WC = waist circumference (centimeters), HADS = Hospital Anxiety and Depression Scale, SD = standard deviation | | |

Appendix B.2. – Alternative index method calculation.

This alternative index has been constructed based on a factor reduction with a principal component analysis using IBM SPSS Statistics (v.23). Missing scores were substituted by the mean. Final scores were transformed following a square-root procedure.

Appendix C.1. –Global brain measures (mm) ANOVA and t-test for groups

| **Mean Cth** | | **Overweight**  **(mean, SD)** | **Range** | **Lean**  **(mean, SD)** | **Range** | **F** | **T** | **p-value** |
| --- | --- | --- | --- | --- | --- | --- | --- | --- |
| Left hemisphere | | 2.56 (0.11) | 2.33 – 2.74 | 2.57 (0.08) | 2.41 – 2.81 | 0.56 | 0.236 | 0.814 |
| Right hemisphere | | 2.54 (0.10) | 2.34 – 2.72 | 2.55 (0.09) | 2.38 – 2.77 | 0.001 | -0.025 | 0.980 |
| Global | | 2.55 (0.10) | 2.33 – 2.73 | 2.56 (0.09) | 2.40 – 2.79 | 0.013 | 0.113 | 0.911 |
|  |  | Cth = cortical thickness (without the effects of age, years of education and gender).  SD = standard deviation | | | | | | |

Appendix C.2. – Cluster-level interactions between overweight and lean participants

|  | **Principal location** | **Extension** | **Size** | **MNI305 coordinates** | | | **Z**  **Value** | **CWP** |
| --- | --- | --- | --- | --- | --- | --- | --- | --- |
|  |  |  |  | **X** | **Y** | **Z** |  |  |
| LH | Pars  triangularis | Pars opercularis, pars triangularis, pars orbitalis, lateral orbitofrontal, insula, precentral, postcentral, paracentral, caudal and rostral middle frontal and superior frontal | 8694.63 | -51.7 | 25.3 | 7.2 | 5.515 | 0.0001 |
|  | Superior  frontal | Superior frontal, caudal and rostral anterior cingulate | 1198.79 | -9.6 | 26.4 | 30.6 | 5.853 | 0.0004 |
|  | Supramarginal | Supramarginal and superior temporal | 1173.16 | -50.9 | -29.8 | 34.8 | 4.825 | 0.0007 |
|  | Inferior  parietal | Inferior parietal and supramarginal | 1288.56 | -47.0 | -59.6 | 39.4 | 4.284 | 0.0001 |
|  | Precuneus | Precuneus and isthmus cingulate | 1042.41 | -4.8 | -59.4 | 26.5 | 3.764 | 0.0010 |
| RH | Precentral | Precentral, paracentral and superior frontal | 1084.21 | 20.7 | -12.2 | 61.4 | 4.952 | 0.0007 |
|  | Precuneus | Precuneus and superior parietal | 1280.42 | 6.8 | -57.7 | 55.7 | 3.951 | 0.0002 |
|  | Transversal  temporal | Transveral temporal, superior temporal and supramarginal | 1009.33 | 50.7 | -17.6 | 5.5 | 3.781 | 0.0019 |
|  | Inferior  parietal | Inferior and superior parietal | 936.82 | 33.1 | -67.6 | 23.2 | 3.505 | 0.0037 |
|  | Lateral  Orbitofrontal | Lateral orbitofrontal, insula and pars triangularis | 936.82 | 28.3 | 29.9 | -9.3 | 3.505 | 0.0054 |
| Principal location based on the peak of maximum intensity. Size is reported in millimiters. CWP = cluster-wise corrected p-value. LH = left hemisphere, RH = right hemisphere. | | | | | | | | |

Appendix D.1. – Alternative index results

There were no significant interactions between groups with a cluster-wise correction (i.e., Monte-Carlo Simulation) set at p < 0.01. With a less restrictive threhold (i.e., p < 0.05) the left precuneus (X= -12.6, Y= -59.9, Z= 25.5, size in mm^2^= 2916.80, Z-value= 3.843, CWP= 0.0001) and the right inferior parietal cortex (X= 37.2, Y= -77.7, Z= 17.2, size in mm^2^= 3001.26, Z-value= 3.837, CWP = 0.001), the right lateral orbitofrontal cortex (X= 13.7, Y= 24.2, Z= -22.1, size in mm^2^= 1871.99, Z-value= 4.443, CWP= 0.004) and the right inferior temporal gyrus (X= 49.1, Y= -9.9, Z= -31.3, size in mm^2^= 1692.14, Z-value= 2.852, CWP= 0.009) showed significant results. The followed trend was the same as in prior results (i.e., overweight/thinning and lean/thickening).

*
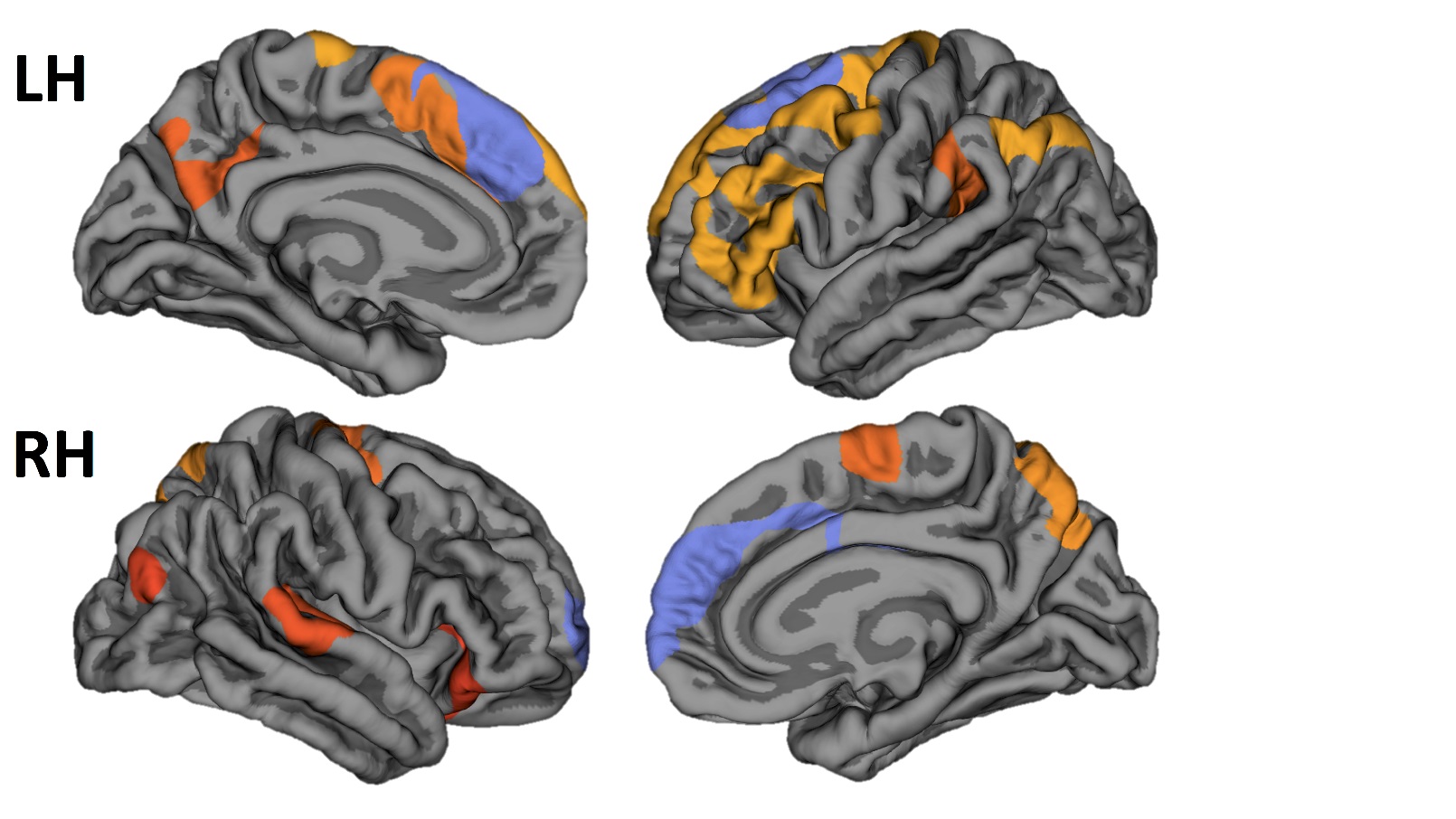
*Appendix D.2. – Overlapping results of group comparisons and interactions with AL

Appendix D.2. – In cold colors (blue), group differences in thickness (lean > overweight) for the left and right frontal superior gyrus (corrected p-value < 0.01). In hot colors, group interactions for the AL index and cortical thickness relationship (corrected p-value < 0.01). LH = Left hemisphere, RH = Right hemisphere.
